# Purification, Characterization and Antioxidant Activities *in Vitro* and *in Vivo* of the Polysaccharides from *Boletus edulis* Bull

**DOI:** 10.3390/molecules17078079

**Published:** 2012-07-05

**Authors:** Aoxue Luo, Aoshuang Luo, Jiandong Huang, Yijun Fan

**Affiliations:** 1 Department of Landscape Plants, Sichuan Agriculture University, Chengdu 611130, China; 2 Chengdu Institute of Biology, Chinese Academy of Sciences, Chengdu 610041, China

**Keywords:** *Boletus edulis* Bull, polysaccharide, purification, antioxidant activity

## Abstract

A water-soluble polysaccharide (BEBP) was extracted from *Boletus edulis* Bull using hot water extraction followed by ethanol precipitation. The polysaccharide BEBP was further purified by chromatography on a DEAE-cellulose column, giving three major polysaccharide fractions termed BEBP-1, BEBP-2 and BEBP-3. In the next experiment, the average molecular weight (Mw), IR and monosaccharide compositional analysis of the three polysaccharide fractions were determined. The evaluation of antioxidant activities both *in vitro* and *in vivo* suggested that BEBP-3 had good potential antioxidant activity, and should be explored as a novel potential antioxidant.

## 1. Introduction

Oxidative stress-induced cell damage triggers both the physiological process of aging and many pathological progressions that can eventually lead to serious health problems [1]. Antioxidants can reduce the cellular oxidative stress by inhibiting the formation of superoxide anions, and by detoxification of reactive oxygen species/reactive nitrogen species through upregulation of cellular defense mechanisms, such as superoxide dismutase, catalase, or glutathione peroxidase [2]. Therefore, research on antioxidants, especially exploration of potent natural compounds with low cytotoxicity from plants, has become an important branch of biomedicine.

Previous studies have indicated that the polysaccharides in plants are not only energy resources, but play key biological roles in many life processes as well. The structure and mechanisms of pharmaceutical effects of bioactive polysaccharides on diseases have been extensively studied, and more natural polysaccharides with different curative effects have been tested and even applied in therapies [3]. Recent researches exhibited that some polysaccharides have been demonstrated to play an important role as free radical scavenger for the prevention of oxidative damage in living organisms [4,5].

*Boletus edulis* Bull is a delicious mushroom that grows in many regions of China, such as Heilongjiang, Henan, Sichuan, Zhejiang, Yunnan, and so on. Modern pharmacological studies demonstrate that it has anti-diabetes and antitumor functions. There are abundant polysaccharides in *Boletus edulis* Bull [6]. Some reported data was found about the crude polysaccharide from *Boletus edulis* [7], but the purification and antioxidant ability *in vitro* and *in vivo* of the polysaccharide from *Boletus edulis* Bull has not been reported, therefore, the purpose of the present investigation was to elucidate the isolation and characterization of water-soluble polysaccharide from *Boletus edulis* Bull, as well as to evaluate its antioxidant activities *in vitro* and *in vivo*.

## 2. Results and Discussion

### 2.1. Isolation and Purification of the Polysaccharides from Boletus edulis Bull

The polysaccharide, named BEBP, was obtained by using the methods of water-extraction and ethanol-precipitation. Before the crude polysaccharide could be obtained, many purification procedures were carried out. For example, the powder of *Boletus edulis* Bull was extracted repeatedly with petroleum ether to remove fat-soluble molecules. Extraction with ethanol can remove the monosaccharides and phenolic compounds and so on. In order to remove these impurities completely, the polysaccharide precipitate was washed successively with petroleum ether, acetone and ethanol. The precipitation procedure was performed repeatedly, and then the residue was dissolved in water and dialyzed against deionized water for 72 h, followed by freeze-drying to yield the polysaccharide. Therefore, through the procedure, the phenolic compounds would be removed from the polysaccharide. In order to confirm the polysaccharides don’t contain any phenolic compounds (exclude acidic phenols), we also detected the content of phenolic compounds by the ferric chloride color method. The result showed the polysaccharides did not contain any phenolic compounds.

**Figure 1:**
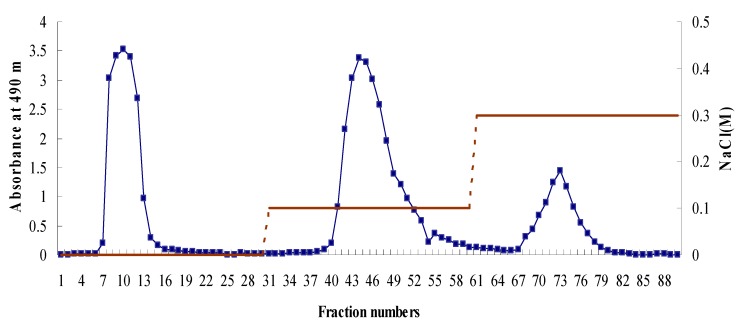
Chromatography of eluted crude polysaccharide (BEBP) on DEAE-Cellulose column (26 mm × 300 mm). BEBP-1 eluted with distilled water; BEBP-2 eluted with 0.1 M NaCl; BEBP-3 eluted with 0.3 M NaCl.

Ion exchange chromatography was performed for purification of the BEBP, and from the DEAE-Cellulose column, BEBP-1 (eluted with water), BEBP-2 (eluted with 0.1 M NaCl) and BEBP-3 (eluted with 0.3 M NaCl) were collected, as shown in Figure 1. Because the molecular weight of polysaccharides is an important factor responsible for biological activities, determining the molecular weight was the first step for the study of the polysaccharides. The molecular weight (Mw) of the polysaccharide fractions BEBP-1, BEBP-2 and BEBP-3 were calculated to be 25.0 KDa, 9.6 KDa and 7.3 KDa, respectively, based on the calibration curve obtained with standard dextrans.

### 2.2. Monosaccharide Compositions of the Polysaccharide Fractions

The monosaccharide compositions of these fractions were analyzed by the trifluoroacetic acid hydrolysis and GC-MS analysis method. The results indicated that mannose and glucose were the major monosaccharides forming the backbone of BEBP-1. The molar ratio of monosaccharide compositions in BEBP-1 was described as follows: glucose/galactose/xylose/mannose/rhamnose = 30.5:6.7:0.8:27.2:1.0. Glucose was the major monosaccharide in BEBP-2, and the molar ratio of glucose/galactose/xylose/mannose was 11.8:3.6:1.0:5.1. BEBP-3 was composed of the mono-saccharides glucose, mannose and galactose in a molar ratio of 7.3:16.6:1.0.

### 2.3. Infrared Spectra Assay

Because the conformations of the polysaccharides are responsible for antioxidant activities, so Fourier transform IR spectrophotometry tests were performed. The FT-IR spectra of the three fractions were presented in Table 1. The fractions exhibited a broad stretching intense characteristic peak at around 3426 cm^−1^ (3415.75–3420.09) for the hydroxyl group and a weak C-H band at around 2929 cm^−1^ (2926.53–2933.55) [8]. The band in 1638–1644 cm^−1^ was due to the bound water [9]. Another specific band appeared in the 1200–1000 cm^−1^ region, a region dominated by ring vibrations overlapped with stretching vibrations of (C-OH) side groups and the (C-O-C) glycosidic band vibrations [10]. The absorption at 853.37 cm^−1^ (BEBP-3) was typical for α-dominating configurations [11] and absorptions at 891.25 cm^−1^ for BEBP-1 and 887.81 cm^−1^ for BEBP-2 were typical for β-dominating configurations [12].

**Table 1:** FT-IR spectra of the polysaccharide fractions.

Samples	Peaks (cm^−1^)	
BEBP-1	3417.33, 2926.53, 1644.91, 1130.37, 891.25	
BEBP-2	3420.09, 2928.16, 1639.03, 1088.76, 887.81	
BEBP-3	3415.75, 2933.55, 1638.69, 1095.54, 853.37	

### 2.4. Antioxidant Activities Analysis

#### 2.4.1. Scavenging Effects of Polysaccharide on Hydroxyl Radicals

**Figure 2:**
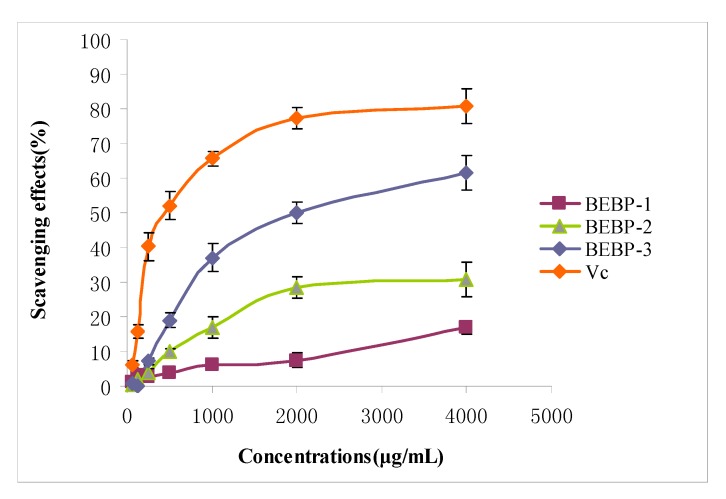
The scavenging effects of different polysaccharide fractions on hydroxyl radical. Results are presented as means ± standard deviations.

The scavenging abilities of different polysaccharide fractions on hydroxyl free radical were shown in Figure 2. The results indicated that the activities of the three samples increased in a concentration dependent manner. Furthermore, the scavenging activities of BEBP-3 increased very significantly with increasing concentrations. Especially at the high dose (4,000 μg/mL), BEBP-3 exhibited very strong activity (61.7%), which was obviously higher than those of BEBP-1 (16.9%) and BEBP-2 (30.9%). Therefore, the results clearly showed that BEBP-3 has potential hydroxyl radical scavenging antioxidant ability.

#### 2.4.2. Scavenging Effects of Polysaccharide on ABTS

**Figure 3:**
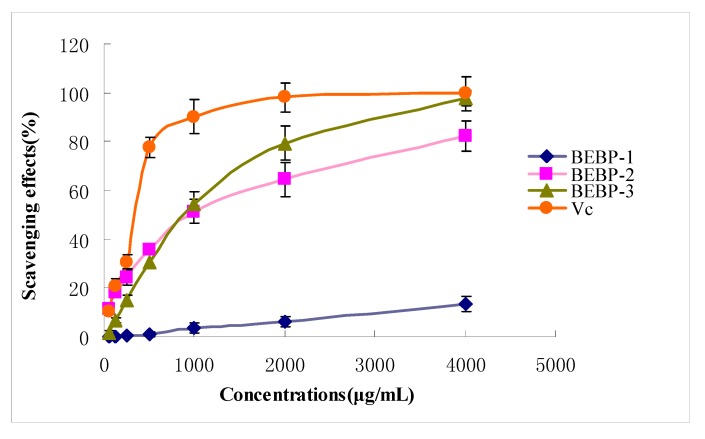
The scavenging effects of polysaccharides on ABTS radical. Results are presented as means ± standard deviations.

The ABTS radical cation decolorization assay is widely applied to evaluate the total antioxidative activity in both lipophilic and hydrophilic samples [13]. The scavenging abilities of various purified polysaccharide fractions (BEBP-1, BEBP-2 and BEBP-3) on ABTS free radical are shown in Figure 3. The three samples exhibited obvious ABTS radical scavenging activities in a concentration-dependent manner. From the Figure, the polysaccharides BEBP-2 and BEBP-3 exhibited an excellent scavenging effect in high doses (from 1,000 to 4,000 μg/mL). At 1,000 μg/mL, the scavenging activity of BEBP-2 was 51.3%. The scavenging effect clearly increased with the dose, and at 4,000 μg/mL, the scavenging effect displayed the highest value (82.3%). The BEBP-3 fraction also revealed an excellent ABTS scavenging activity, especially at 4,000 μg/mL, where the effect reached 97.7%. On the other hand, the scavenging activity on ABTS of BEBP-1 was weak, even at the high dose of 4,000 μg/mL, and the scavenging effect was only 13.4%. From the figure, the ABTS radical scavenging ability decreased in the order of BEBP-3 > BEBP-2 > BEBP-1. Therefore, the results indicated that BEBP-3 had strong scavenging power for ABTS radicals and should be explored as a novel potential antioxidant substance. 

#### 2.4.3. Effect of the Polysaccharides on Reducing Power

**Figure 4:**
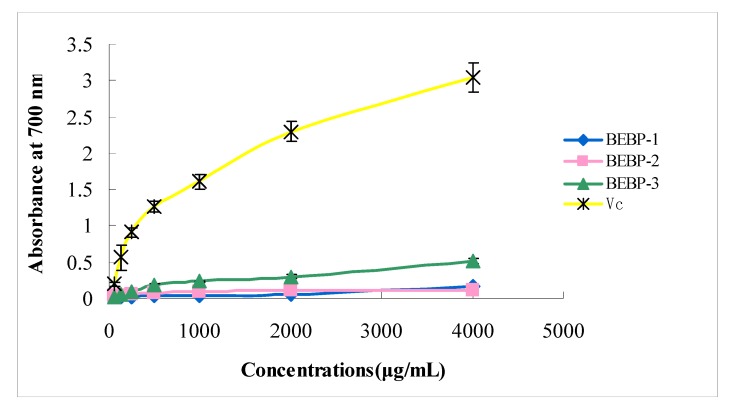
Effect of the polysaccharides on reducing power. Results are presented as means ± standard deviations.

Research has revealed that there is a direct correlation between antioxidant activities and reducing power [14]. In order to measure the reducing power of different polysaccharide fractions, the Fe^3+^-Fe^2+^ transformation in the presence of samples of various concentrations was investigated. The reducing capabilities of three polysaccharide fractions are presented in Figure 4. A concentration-dependent reducing power of the three samples was again identified. All extracts showed low reducing power at the low doses (from 62 to 1,000 μg/mL). At a high concentration of 1,000–4,000 μg/mL, BEBP-1 and BEBP-2 also exhibited low reducing powers, however, the reducing effect of BEBP-3 was higher than that of BEBP-1 and BEBP-2 at the high dose. None of the three samples showed significant reducing power compared to Vitamin C (Vc).

#### 2.4.4. Antioxidant Activity *in Vivo*

**Figure 5:**
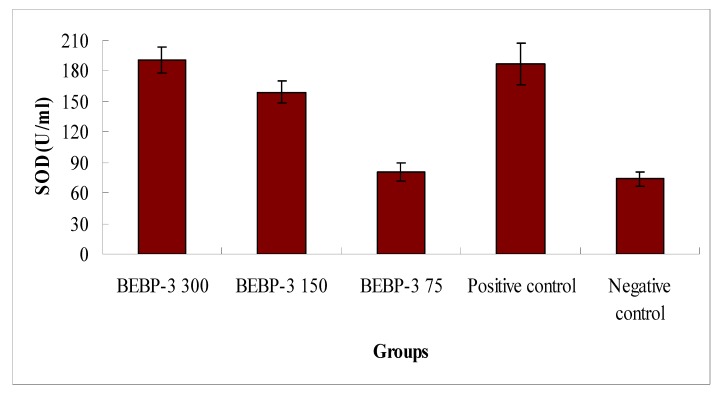
SOD activity analysis in mice. Results are presented as means ± standard deviations. The positive control is Vitamin C at the concentration of 150 mg/kg.

According to the results above, the effects of antioxidant activities *in vitro* of BEBP-1 and BEBP-2 were weak. On the other hand, BEBP-3 exhibited strong free radical scavenging effects, therefore, in order to investigate in-depth the antioxidant activity of the polysaccharide fraction BEBP-3, its antioxidant activity *in vivo* was tested. The results are shown in Figure 5. SOD activities of different doses of BEBP-3 exhibited dose-dependent behavior. At 150 mg/kg, BEBP-3 exhibited high SOD activity, and the SOD activity value of BEBP-3 was 159.27 U/mL, which was close to that of the positive control (Vitamin C). At the high dose of 300 mg/kg, particularly, SOD activity of BEBP-3 was 190.3 U/mL, which was higher than that of vitamin C (*p* < 0.05). However, the SOD activity at low concentrations was much less evident, which is similar to that of the negative control. The results were therefore an indication of enhancement SOD activity of BEBP-3 for high concentrations.

**Figure 6:**
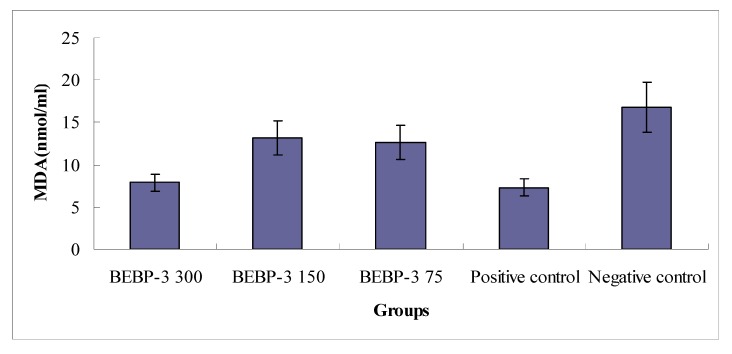
Determination of MDA contents in blood serum from the mice. Results are presented as means ± standard deviations. The positive control is Vc (150 mg/kg).

The MDA value was estimated according to the thiobarbituric acid (TBA) method [15]. The concentrations of MDA in blood serum from the mice were determined with a MDA Assay Kit. Briefly, the samples added with TBA were heated in an acidic environment, then, the absorbance of the resulting solution was measured at 532 nm. The results in Figure 6 exhibit a significant pattern of a decreasing MDA concentration in blood serum with increasing BEBP-3 concentration. At 300 mg/kg, the concentration of MDA was 7.9 nmol/mL, close to that of the positive control (7.3 nmol/mL) (*p* < 0.05). This can be interpreted as a significant effect of BEBP-3 at high concentrations on MDA scavenging *in vivo*.

## 3. Experimental

### 3.1. Materials and Chemicals

Dextrans of different molecular weights were purchased from Pharmacia Co. (Uppsala, Sweden). The standard monosaccharides (glucose, mannose, rhamnose, galactose, xylose and arabinose) were purchased from the Chinese Institute for the Control of Pharmaceutical and Biological Products (Beijing, China). 2,2-Azinobis-6-(3-ethylbenzothiazoline sulfonic acid (ABTS) radical was purchased from Merck Co. (Darmstadt, Germany). DEAE-cellulose, 1,1-diphenyl-2-picrylhydrazyl (DPPH) radical and Vitamin C (Vc) were purchased from Sigma (St. Louis, MO, USA). Superoxide dismutase (SOD) Assay Kit001 and Malondialdehyde (MDA) Assay Kit A003 were purchased from the Institute of Biological Engineering of Nanjing Jianchen (Nanjing, China). Trifluoroacetic acid (TFA), pyridine, methanol, and acetic acid, ethanol, acetic anhydride and all other chemicals and reagents were analytical grade.

### 3.2. Extraction and Purification the Polysaccharides of *Boletus edulis* Bull

Extraction and purification of the polysaccharides were carried out according to the method of Luo *et al.* [16], with some modifications. The dried fruiting bodies (100 g) of *Boletus edulis* Bull were crushed and then extracted with petroleum ether for two hours, and further extracted with 80% ethanol at 90 °C for 2 h. After filtering, the residue was further extracted three times with double-distilled water at 100 °C for 2 h. Then all extracts were combined, concentrated using a rotary evaporator at 55 °C and filtered. The extract was deproteinized three times using the Sevag reagent [17], and the polysaccharide was determined to be free of proteins as scanning its UV spectra in 280 nm. After removal of the Sevag reagent, the extract was precipitated by adding ethanol (four times the volume of aqueous extract), and the mixture was kept overnight at 4 °C to yield the polysaccharide. The precipitate was collected by centrifugation at 4,000 rpm for 10 min, washed successively with petroleum ether, acetone and ethanol, and the procedure of precipitation was performed repeated, and then dissolved in water and dialyzed against deionized water for 72 h, freeze-drying to yield the crude polysaccharide, which was named BEBP. The polysaccharide (BEBP) was redissolved in deionized water and then applied to a column (300 × 26 mm) of DEAE-cellulose. After loading with sample, the column was eluted by deionized water, 0.1 M and 0.3 M NaCl respectively, at a flow rate of 1.0 mL/min. Fractions (8 mL) were collected by a fraction collector. All of these fractions were analyzed for the carbohydrate content by the phenol-sulfuric acid assay [18]. The chromatography profile was drawn by Microsoft Excel 2000. The peak with the highest polysaccharide content was collected, dialyzed and then freeze-dried.

### 3.3. Molecular Weight Determination

The molecular weights of the polysaccharides were determined by the Gel Permeation Chromatography (GPC) method, which has been described by Yamamoto *et al.* [19], in combination with a Waters HPLC (Waters 515, Milford, MA, USA) equipped with a Ultrahydrogel Linear Column (300 × 7.8 mm). The column was eluted with 0.2 M phosphate buffer (pH 7.0) at a flow rate of 0.7 mL/min and detected by a Waters 2410 refractive index detector (RID). Dextran standards with different molecular weights (2500, 4600, 7100, 10,000, 21,400, 41,100, 84,400, 133,800, 200,000 Da) were used to plot the calibration curve.

### 3.4. Analysis of Monosaccharide Compositions

GC-MS was used for identification and quantification of the monosaccharides from *Boletus edulis* Bull. First, the polysaccharide (10.0 mg) was hydrolyzed with 2.0 M TFA at 110 °C for 4 h in a sealed glass tube. Then the hydrolysate was evaporated to dryness and dissolved in 0.5 mL of pyridine, after 10.0 mg hydroxylamine hydrochloride and 2.0 mg myo-inositol (as internal reference) were added to the solution, it was allowed to react at 90 °C for 30 min. The tube was cooled to room temperature, and then 0.5 mL of acetic anhydride was added and mixed thoroughly by vortexing. The tube was sealed and incubated in a water bath shaker set at 90 °C for 30 min. After cooled, approximately 1.0 μL of clear supernatant was loaded onto an Rtx-5SilMS column (30 m × 0.32 mm × 0.25 μm) of the GC-MS. Alditol acetates of authentic standards (glucose, mannose, rhamnose, galactose, xylose and arabinose) with myo-inositol as the internal standards were prepared and subjected to GC-MS analysis separately in the same way [20,21].

### 3.5. Infrared Spectra Analysis

The structural characteristics of the polysaccharides fractions were determined on a Fourier transform IR spectrophotometer (Perkin-Elmer Corp., Waltham, MA, USA). The purified polysaccharides were ground with KBr powder and then pressed into pellets for IR measurements in the frequency range of 4000–500 cm^−1^ [22].

### 3.6. Assays for Antioxidant Activities

#### 3.6.1. Hydroxyl Radical Scavenging Assay

The hydroxyl radical scavenging activities of the purified polysaccharides were measured according to the methods of Wang *et al.* [23] and Luo *et al.* [24], with some modifications. Briefly, samples of different concentrations (4000, 2000, 1000, 500, 250, 125, 62.5 μg/mL) were incubated with 2.0 mmol/L EDTA-Fe (0.5 mL), 3% H_2_O_2_ (1.0 mL) and 0.36 mg/mL crocus in 4.5 mL sodium phosphate buffer (150 mM, pH 7.4) for 30 min at 37 °C and hydroxyl radical was detected by monitoring absorbance at 520 nm. The hydroxyl radical scavenging effect was calculated as: 



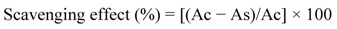



where As is the A_520_ of sample and Ac is the A_520_ of control. In the control, sample was substituted with distilled water, and sodium phosphate buffer replaced H_2_O_2_.

#### 3.6.2. ABTS Radicals Scavenging Assay

The ABTS assay is often used in evaluating total antioxidant power of single compounds and complex mixtures of various plants. The radical scavenging activity of the purified polysaccharide fractions against ABTS radical cation was measured using the methods of Re *et al.* [25] and Luo *et al.* [26], with some modifications. ABTS was produced by reacting 7 mmol/L of ABTS solution with 2.45 mmol/L of potassium persulphate, and the mixture was kept in the dark at room temperature for 16 h. At the moment of use, the ABTS solution was diluted with ethanol to an absorbance of 0.70 ± 0.02 at 734 nm. The samples (0.2 mL) with various concentrations (4,000, 2,000, 1,000, 500, 250, 125, 62.5 μg/mL) were added to 2 mL of ABTS^+^ solution and mixed vigorously. After reaction at room temperature for 6 min, the absorbance at 734 nm was measured. The ABTS^+^ scavenging effect was calculated by the following formula: 



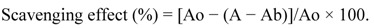



where Ao: A_734_ of ABTS without sample, A: A_734_ of sample and ABTS, and Ab: A_734_ of sample without ABTS.

#### 3.6.3. Reducing Power

The reducing powers of the purified polysaccharide fractions were measured by the methods of Fan *et al.* [27] and Yen *et al.* [28], with some modifications. Purified polysaccharides of a variety of concentrations (4,000, 2,000, 1,000, 500, 250, 125, 62.5 μg/mL) were tested. The sample (1.0 mL) was first mixed with a phosphate buffer (volume 2.5 mL, concentration 0.2 mol/L, pH 6.6) and potassium ferricyanide [K_3_Fe(CN)_6_] (volume 2.5 mL, 1%). The mixture was then incubated at 50 °C for 20 min. The reaction was terminated by a 2.5 mL TCA solution (0.1%) and the resulting mixture was centrifuged at 3,000 rpm for 10 min. The supernatant (2.5 mL) was mixed with 2.5 mL of distilled water and 0.5 mL of ferric chloride (concentration 6 mmol/L).The absorbance of the obtained material was measured at 700 nm. It was anticipated that increased reducing power would be associated with increased absorbance of the test mixture.

#### 3.6.4. Antioxidant Activity *in Vivo*

Kunming mice (provided by Sichuan Academy of Medical Science, Chengdu, Sichuan, China), weighing in the range of 18 to 22 g, were kept in separated cages at a temperature of 21 ± 1 °C and a 50 to 60% of relative humidity [29]. They underwent 12-h light-and-dark cycles with free access to food and water. A total of 50 mice were evenly and randomly divided into five groups, including a D-galactose model control group (negative control), a vitamin C (150 mg/kg) group (positive control), and dose-dependent polysaccharide groups (300, 150, and 75 mg/kg body weight). Each group was induced by a single intraperitoneal injection of D-galactose (150 mg/kg/day body weight, dissolved in a 0.9% saline solution) [30]. The mice in the D-galactose model control group were given a 0.2-mL physiological saline solution (0.9% w/v) once daily for 20 consecutive days by intraperitoneal injection. Twenty-four hours after the last drug administration, blood samples were obtained from the eyepit of the mice and processed for serum. The superoxide dismutase (SOD) activity and the malondialdehyde (MDA) level were also measured [31].

### 3.7. Statistical Analysis

The data were presented as mean ± standard deviation. Statistical analysis was conducted with the SPSS 16.0 software package.

## 4. Conclusions

Anion-exchange and size-exclusion chromatography were used to prepare the purified polysaccharide samples. From the results above, it was concluded that the water-extracted crude polysaccharide BEBP from *Boletus edulis* Bull could be purified by DEAE-cellulose column chromatography. Three major polysaccharide fractions (BEBP-1, BEBP-2 and BEBP-3) were obtained, and the polysaccharide fractions prepared were confirmed to be of high purity. Antioxidant tests indicated that BEBP-1 and BEBP-2 have no significant effects, but BEBP-3 exhibited a powerful scavenging effect on hydroxyl radicals and ABTS radical. In *in vivo* assays, the polysaccharide BEBP-3 was found to increase the levels of antioxidant enzymes SOD and to decrease the MDA content in blood serum. It was confirmed that BEBP-3 could protect tissues against oxidative damage. Enhanced SOD activity in mice blood serum also can be related to the *in vivo* antioxidant activity of BEBP-3. With such strong antioxidant ability, BEBP-3 was identified as a potential antioxidant.
